# Critical role of oxidized LDL receptor-1 in intravascular thrombosis in a severe influenza mouse model

**DOI:** 10.1038/s41598-021-95046-y

**Published:** 2021-08-03

**Authors:** Marumi Ohno, Akemi Kakino, Toshiki Sekiya, Naoki Nomura, Masashi Shingai, Tatsuya Sawamura, Hiroshi Kida

**Affiliations:** 1 Laboratory for Biologics Development, International Institute for Zoonosis Control, Hokkaido University, Kita 20 Nishi 10, Kita-ku, Sapporo, 001-0020 Japan; 2 Department of Molecular Pathophysiology, School of Medicine, Shinshu University, Matsumoto, Japan

**Keywords:** Influenza virus, Coagulation system

## Abstract

Although coagulation abnormalities, including microvascular thrombosis, are thought to contribute to tissue injury and single- or multiple-organ dysfunction in severe influenza, the detailed mechanisms have yet been clarified. This study evaluated influenza-associated abnormal blood coagulation utilizing a severe influenza mouse model. After infecting C57BL/6 male mice with intranasal applications of 500 plaque-forming units of influenza virus A/Puerto Rico/8/34 (H1N1; PR8), an elevated serum level of prothrombin fragment 1 + 2, an indicator for activated thrombin generation, was observed. Also, an increased gene expression of oxidized low-density lipoprotein (LDL) receptor-1 (*Olr1*), a key molecule in endothelial dysfunction in the progression of atherosclerosis, was detected in the aorta of infected mice. Body weight decrease, serum levels of cytokines and chemokines, viral load, and inflammation in the lungs of infected animals were similar between wild-type and *Olr1* knockout (KO) mice. In contrast, the elevation of prothrombin fragment 1 + 2 levels in the sera and intravascular thrombosis in the lungs by PR8 virus infection were not induced in KO mice. Collectively, the results indicated that OLR1 is a critical host factor in intravascular thrombosis as a pathogeny of severe influenza. Thus, OLR1 is a promising novel therapeutic target for thrombosis during severe influenza.

## Introduction

Influenza is a respiratory disease and remains a major health concern, causing approximately half a million deaths per year globally^1^. Influenza virus, a causative pathogen of influenza, infects and proliferates in the epithelial cells of respiratory tissues. In response to virus proliferation, host innate immunity is induced and triggers proinflammatory responses^2^. Although the detailed mechanisms of influenza pathogenesis are poorly understood, severe influenza is characterized by tissue edema and single- or multiple-organ dysfunction, one of the lethal pathological conditions^3,4^. Given the previous findings on sepsis-induced organ dysfunction^5^, microvascular thrombosis is considered to contribute to tissue injury and multiple-organ dysfunction syndrome in severe influenza. In fact, an imbalance between coagulation and fibrinolysis during severe influenza has been confirmed in mouse and ferret models^6,7^. Furthermore, the increased risk of acute myocardial infarction and venous thromboembolism after acute infectious diseases, including influenza, and the prolonged prothrombin time (PT) in human clinical cases further suggest that abnormal blood coagulation caused by viral infection also occurs in humans^4,8–10^. Therefore, the elucidation of the detailed mechanisms of abnormal blood coagulation during severe influenza would provide novel and important insights into the further understanding of influenza pathogenesis.

Oxidized low-density lipoprotein (LDL) receptor-1 (OLR1) is originally identified as a receptor for oxidized LDL in vascular endothelial cells^11^. Previous studies have demonstrated that OLR1 mediates vascular endothelial cell damage and atherosclerosis and the enhancement of vascular permeability caused by oxidized LDL^12,13^. Furthermore, a wide range of physiological functions of OLR1 far beyond cardiovascular diseases has been elucidated, for example, in the regulation of inflammatory responses to endotoxin^14,15^. In a lung injury mouse model, intraperitoneal injection of endotoxin rapidly increases OLR1 protein expression in the lungs, and induced OLR1 activates inflammatory nuclear factor-κB (NF-κB) signaling, leukocyte accumulation, and hyperpermeability in the lungs^15^. Also, OLR1 expressed in vascular endothelial cells promotes the attachment of activated platelets to the cells and endothelial dysfunction^16,17^, which could change the vascular wall into prothrombotic. These previous findings led to the hypothesis that OLR1 is a bridge between inflammation and abnormal blood coagulation and plays an important role in influenza pathogenesis.

This study evaluated influenza-associated abnormal blood coagulation utilizing a severe influenza mouse model that was previously established^18^. Furthermore, blood coagulation profiles were compared between wild-type (WT) and *Olr1* global knockout (KO) mice to investigate the roles of OLR1 as a host factor in cytokine production, coagulation abnormality, and lung intravascular thrombosis during influenza.

## Results

### Activated thrombin generation in a severe influenza mouse model

The effect of influenza virus infection on the blood coagulation system of the host was investigated in a severe influenza mouse model, in which weight loss of 25% or more, a criterion for a humane endpoint, is induced within 7 days of infection^18^. PR8 virus was intranasally infected to male mice at a dose of 500 plaque-forming units (PFUs)/mouse, and samples were collected at 1, 3, and 6 days post-infection (dpi). Body weight changes at each sampling point were 99.9% ± 0.4% in control mice and 99.8% ± 0.5% in infected mice at 1 dpi, 101.1% ± 0.7% in control mice and 86.1% ± 0.4% in infected mice at 3 dpi, and 101.5% ± 0.9% in control mice and 74.0% ± 0.7% in infected mice at 6 dpi. Significant body weight loss was observed in infected mice, compared to control mice, at 3 and 6 dpi [*p* < 0.0001, two-way analysis of variance (ANOVA)]. In addition to body weight loss, infected mice showed ruffled fur, lower motor activity, and dehydration at 6 dpi. Measurements of blood coagulation parameters were performed with whole-blood, and sera were collected at 1, 3, and 6 dpi for samples at a very early stage, the onset of a symptom, and the lethal phase during influenza, respectively.

**Figure 1:**
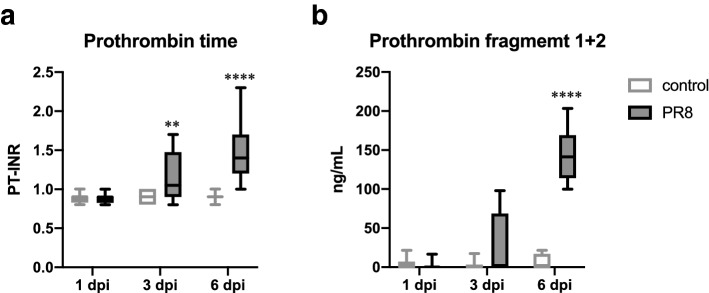
Blood coagulation parameters in control versus PR8 virus-infected mice at 1, 3, and 6 dpi. Mice were intranasally inoculated with PBS control or PBS comprising PR8 virus, and whole-blood and serum samples were collected for the measurement of (**a**) PT-INR [n = 11 (PR8-6 dpi group) or 12 (the other groups)] and (**b**) prothrombin fragment 1 + 2 [n = 6 (PR8-6 dpi group) or 8 (the other groups)], respectively, at 1, 3, and 6 dpi. (**a** and **b**) Values are represented by box-and-whiskers plots as follows: the central line in the box is the median, the bottom and top lines of the box are the first and third quartiles, respectively, whiskers are the minimum to maximum values. In each panel, white and gray boxes indicate data from control and PR8 virus-infected mice at 1, 3, and 6 dpi, respectively. **p < 0.01, ****p < 0.0001, two-way ANOVA using a multiple-comparison correction, control versus PR8 virus-infected mice at each time point. PT-INR, international normalized ratio of prothrombin time; PR8, influenza virus A/Puerto Rico/8/34; dpi, days post-infection.

The international normalized ratio of PT (PT-INR) and prothrombin fragment 1 + 2 concentrations were examined as blood coagulation parameters. As shown in Fig. 1a, the PT-INR level, a clinical index of the duration of blood coagulation, was elevated in PR8 virus-infected mice at 3 and 6 dpi (0.90 ± 0.03 in control mice and 1.16 ± 0.09 in infected mice at 3 dpi, *p* < 0.05, two-way ANOVA; 0.89 ± 0.02 in control mice and 1.46 ± 0.11 in infected mice at 6 dpi; *p* < 0.0001, two-way ANOVA). A time-dependent elevation of the PT-INR level in the host with severe influenza was also indicated (*p* < 0.0001, two-way ANOVA). Moreover, the serum level of prothrombin fragment 1 + 2, a specific indicator of activated thrombin generation, drastically increased in infected mice (Fig. 1b). The levels were 3.89 ± 2.79 ng/mL in control mice and 2.08 ± 2.08 ng/mL in infected mice at 1 dpi, 2.66 ± 2.17 ng/mL in control mice and 26.04 ± 14.48 ng/mL in infected mice at 3 dpi, and 5.77 ± 3.48 ng/mL in control mice and 143.77 ± 14.62 ng/mL in infected mice at 6 dp. A significant difference between control and infected mice was observed only in samples collected at 6 dpi (*p* < 0.0001, two-way ANOVA). These results clearly showed that abnormal blood coagulation demonstrated by both prolonged PT and increased thrombin generation from prothrombin is induced in the host at the lethal phase of severe influenza. On the other hand, PR8 virus infection at 25 PFU did not induce the elevation of serum prothrombin fragment 1 + 2 or PT-INR in 6 days in our preliminary experiment. Therefore, subsequent experiments were conducted using lethal infection conditions of 500 PFU to investigate blood coagulation abnormalities in severe influenza.

### The elevation of expression of *Il6*, *Icam1*, and *Olr1* genes in the aorta and lungs of mice during severe influenza

**Figure 2:**
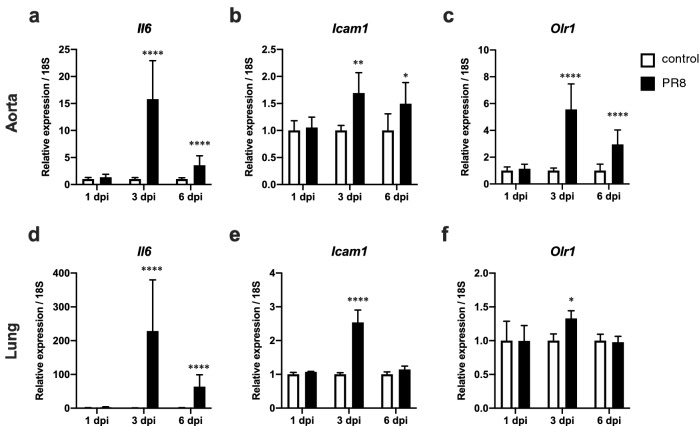
Expression of inflammation-related genes in the aorta and lungs. Mice were intranasally inoculated with PBS control or PBS comprising PR8 virus, and (**a–c**) aortic and (**d–f**) lung samples were collected at 1, 3, and 6 dpi. (**a** and **d**) *Il6* , (**b** and **e**) *Icam1*, and (**c** and **f**) *Olr1* gene expression was normalized with that of 18S from real-time PCR analyses. (**a–f**) Gene expression of PR8 virus-infected mice is presented as the fold changes relative to those of control mice at each time point. Bars represent the mean ± SEM of 11(PR8-3 dpi and PR8-6 dpi groups) or 12 (the other groups) animals for aorta and 4 animals for lung, respectively. White and black bars indicate data from control and PR8 virus-infected mice, respectively. * *p* < 0.05, **p < 0.01, ****p < 0.0001, two-way ANOVA using a multiple-comparison correction, control versus PR8 virus-infected mice at each time point. PR8, influenza virus A/Puerto Rico/8/34; dpi, days post-infection.

Endothelial dysfunction was considered involved in the induction of abnormal blood coagulation during severe influenza. Thoracic aorta and lung samples from control and PR8 virus-infected mice were collected at 1, 3, and 6 dpi, and gene expression related to inflammation and endothelial functions was investigated (Fig. 2). *Interleukin-6* (*Il6*), a proinflammatory cytokine, was significantly increased by 15.8- and 3.6-fold in the aorta (Fig. 2a) and by 228.46- and 63.78-fold in the lung (Fig. 2d) of infected mice at 3 and 6 dpi, respectively (*p* < 0.0001, two-way ANOVA). *Intercellular adhesion molecule-1* (*Icam1*), which encodes adhesion molecules for leukocytes, was expressed at slightly but significantly increased levels in the infected mouse aorta (1.69- and 1.49-fold at 3 and 6 dpi, respectively, *p* < 0.05, two-way ANOVA; Fig. 2b), whereas its expression in the lungs was significantly increased only at 3 dpi (2.53-fold, *p* < 0.0001, two-way ANOVA; Fig. 2e). OLR1, an endothelial receptor for LDL, is a key player in oxidized LDL-induced atherogenesis and endotoxin-induced inflammation^14,19^. Interestingly, aortic *Olr1* expression was significantly increased in PR8 virus-infected mice by 5.6- and 3.0-fold at 3 and 6 dpi, respectively (*p* < 0.0001, two-way ANOVA; Fig. 2c). The lung of infected mice also showed a significant level of induction of *Olr1* at 3 dpi (1.33-fold, *p* < 0.05, two-way ANOVA; Fig. 2f). Also, expression levels of *Il6* and *Olr1* were significantly correlated in samples collected at 3 dpi (aorta, R^2^ = 0.8585, *p* < 0.0001; lung, R^2^ = 0.6488, *p* < 0.05; linear regression analysis). Aortic samples collected at 6 dpi also showed a weaker but significant correlation between the levels of these genes (R^2^ = 0.4516, *p* < 0.0005, linear regression analysis). Given its critical role in endotoxin-induced inflammation and endothelial dysfunction^14,17^, OLR1 was hypothesized to be involved in local and systemic inflammation as well as abnormal blood coagulation observed in mice with severe influenza. This hypothesis led to conduct influenza virus infection experiments in *Olr1* KO (KO) mice, which was previously established^19^, to confirm whether this host factor is involved in those pathological events.

### Similar body weight loss and virus titer in the lungs of *Olr1* KO mice upon influenza virus infection to those in WT mice

**Figure 3:**
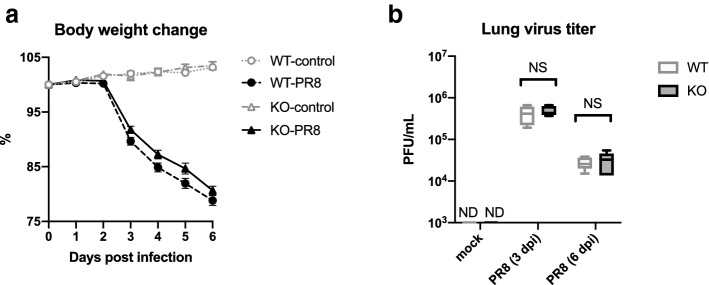
Effect of OLR1 on virus infection-induced body weight decrease and lung viral replication. WT and KO mice were intranasally inoculated with PBS control or PBS comprising PR8 virus, and (**a**) body weight change and (**b**) lung virus titers were evaluated. (**a**) The body weight change of mice was calculated as a percentage of the original weight. Symbols represent mean ± SEM (WT-control, n = 9; WT-PR8, n = 9; KO-control, n = 6; KO-PR8, n = 8). Circles and triangles indicate data from WT and KO mice, respectively. Open and closed symbols indicate PBS control and PR8 virus-infected mice, respectively. No significant difference was detected between WT and KO mice at each time point by two-way ANOVA. (**b**) At 3 and 6 dpi, mice were euthanized for the collection of lung samples, and plaque assays on MDCK cells were performed to calculate the lung viral titers in each sample (n = 5). Values are represented by box-and-whiskers plots as follows: the central line in the box is the median, the bottom and top lines of the box are the first and third quartiles, respectively, whiskers are the minimum to maximum values. White and gray boxes indicate data from WT and KO mice, respectively. No significant difference was detected between WT and KO mice in each treatment group by two-way ANOVA. (**a** and **b**) PR8, influenza virus A/Puerto Rico/8/34; PFU, plaque-forming unit; dpi, days post-infection; WT, wild type mice; KO, *Olr1* knockout mice; ND, not detected; NS, not significant.

PR8 virus was intranasally infected to WT and KO mice at a dose of 500 PFU/mouse. Blood coagulation parameters and serum cytokine levels as well as the histopathological changes in the lungs were also evaluated. Both mice showed significant body weight losses from 3 dpi onward (*p* < 0.0001, two-way ANOVA; Fig. 3a). At 6 dpi, body weight changes were 103.2% ± 0.2% in WT-control mice, 78.8% ± 0.9% in WT-PR8 virus-infected mice, 103.5% ± 0.7% in KO-control mice, and 80.7% ± 0.8% in KO-PR8 virus-infected mice. No significant difference was detected in body weight losses between WT and KO mice at any time points (*p* > 0.05, two-way ANOVA). All infected mice were euthanized at 6 dpi for a humane endpoint indexed by weight loss, but the hair gloss looked better in KO mice, and they were more active than WT mice. The average lung virus titers of WT and KO mice at 3 and 6 dpi were 4.16 × 10^5^ ± 0.90 × 10^5^ in WT mice and 4.94 × 10^5^ ± 0.58 × 10^5^ in KO mice at 3 dpi and 2.78 × 10^4^ ± 0.41 × 10^4^ in WT mice and 3.04 × 10^4^ ± 0.77 × 10^4^ in KO mice at 6 dpi (Fig. 3b). No significant difference was detected between WT and KO at both time points ((*p* > 0.05, two-way ANOVA). These results indicated that the absence of OLR1 did not affect the infection and replication of the virus in the lungs, at least under this experimental condition.

### Restored blood coagulation abnormalities during severe influenza in *Olr1* KO mice

**Figure 4:**
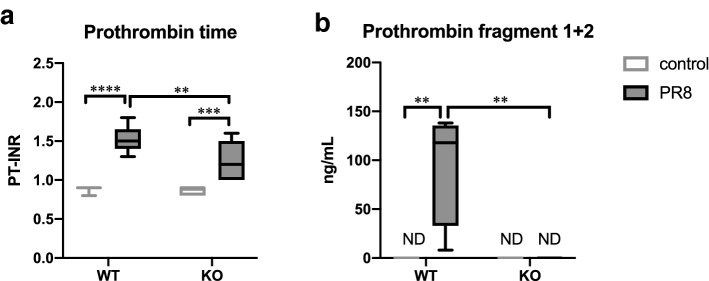
Effect of OLR1 on virus infection-induced blood coagulation abnormalities. WT and KO mice were intranasally inoculated with PBS control or PBS comprising PR8 virus, and whole-blood and serum samples were collected for the measurement of (**a**) PT-INR (WT control, n = 9; WT PR8, n = 9; KO control, n = 7; KO PR8, n = 7) and (**b**) prothrombin fragment 1 + 2 (n = 4), respectively, at 6 dpi. (**a** and **b**) Values are represented by box-and-whiskers plots as follows: the central line in the box is the median, the bottom and top lines of the box are the first and third quartiles, respectively, whiskers are the minimum to maximum values. In each panel, white and gray boxes indicate data from control and infected mice, respectively. **p < 0.01, ***p < 0.0005, ****p < 0.0001, two-way ANOVA using a multiple-comparison correction. PR8, influenza virus A/Puerto Rico/8/34; WT, wild type mice; KO, *Olr1* knockout mice; ND, not detected.

Upon PR8 virus infection, the PT-INR level was significantly elevated from 0.89 ± 0.01 to 1.53 ± 0.05 in WT mice (*p* < 0.0001, two-way ANOVA) and 0.87 ± 0.02 to 1.23 ± 0.09 in KO mice (*p* < 0.0005, respectively, two-way ANOVA) in Fig. 4a. When comparing WT and KO mice, a significant difference in the infection-induced PT-INR level was detected (*p* < 0.01, two-way ANOVA). In contrast, elevated serum prothrombin fragment 1 + 2 was observed only in infected WT mice (95.524 ± 29.8 ng/mL), whereas the value was not altered in KO mice after virus infection (Fig. 4b). These results indicated that the host factor OLR1 plays an important role in pathological blood coagulation during severe influenza. Particularly, thrombin generation from prothrombin in mice with severe influenza was considered critically regulated by OLR1.

### Similar cytokine responses in *Olr1* KO mice upon influenza virus infection to those in WT mice

**Figure 5:**
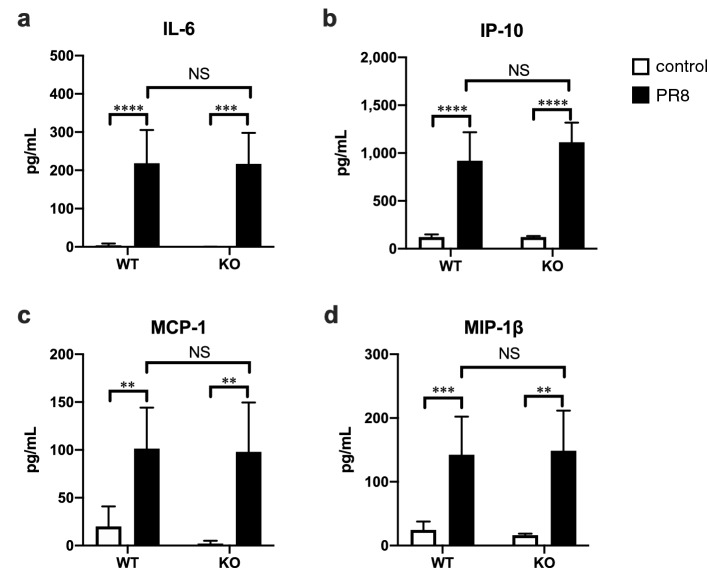
Effect of OLR1 on virus infection-induced systemic inflammation. WT and KO mice were intranasally inoculated with PBS alone or PBS comprising PR8 virus, and serum samples were collected at 6 dpi. Serum levels of (**a**) IL-6, (**b**) IP-10, (**c**) MCP-1, and (**d**) MIP-1β were measured by a multiplex assay. (**a–d**) Bars represent the mean ± SEM of 3 (KO control group) or 8 (the other groups) animals. In each panel, white and black bars indicate data from control and PR8 virus-infected mice, respectively. **p < 0.01, ***p < 0.005, ****p < 0.0001, two-way ANOVA using a multiple-comparison correction. WT, wild type mice; KO, *Olr1* knockout mice; NS, not significant.

In contrast, influenza virus infection-induced systemic inflammation was not affected by the absence of OLR1 (Fig. 5). Proinflammatory cytokine and chemokine IL-6 (Fig. 5a), interferon-γ-induced protein-10 (IP-10; Fig. 5b), monocyte chemoattractant protein-1 (MCP-1; Fig. 5c), and macrophage inflammatory protein-1β (MIP-1β; Fig. 5d) were detected in PR8 virus-infected KO mice at very similar levels to those in infected WT mice. For example, the serum IL-6 levels were 3.68 ± 1.73 pg/mL in WT-control mice, 218.4 ± 32.9 pg/mL in WT-PR8 virus-infected mice, 0.39 ± 0.24 pg/mL in KO-control mice, and 217.0 ± 28.7 pg/mL in KO-PR8 virus-infected mice. Two-way ANOVA demonstrated only effects of virus infection on all cytokines and chemokines (*p* < 0.0001) but not in the presence or absence of OLR1 (*p* > 0.05). Consistently, there was no difference in the induced levels of *Il6* gene expression in the aorta and lungs between WT and KO mice (Supplemental Fig. S1). The results further suggested that OLR1 is dispensable for local cytokine production. Collectively, these results revealed that OLR1 plays a role in the activation of thrombin generation in severe influenza without affecting the production of inflammatory cytokines.

### Suppression of the influenza-induced thrombosis in the lungs of *Olr1* KO mice

**Figure 6:**
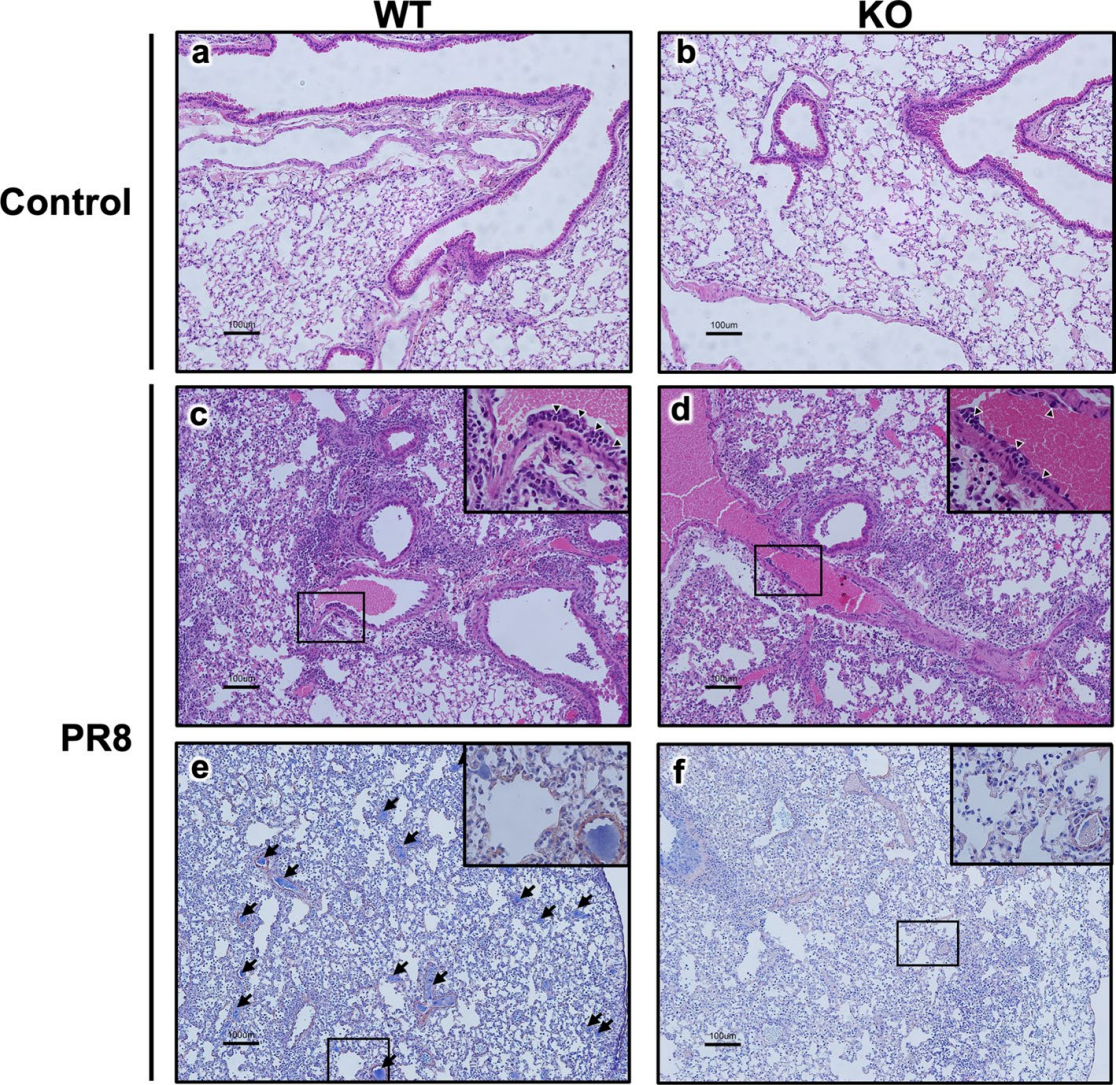
Effect of OLR1 on virus infection-induced inflammation and intravascular thrombosis in the lungs. WT and KO mice were intranasally inoculated with PBS alone or PBS comprising the PR8 virus. Lung samples were collected at 6 dpi, fixed in 4% paraformaldehyde, embedded in paraffin, and cut in 5 μm. For histopathological analyses, sections were stained with (**a–d**) HE or (**e** and **f**) PTAH. (**a**) WT control, (**b**) KO control, (**c** and **e**) WT infected with PR8 virus, and (**d** and **f**) KO infected with PR8 virus. Scale bars, 100 μm. (**c** and **d**) Arrowheads in inserts indicate the leukocytes in the vascular intima. (**e** and **f**) Arrows indicate the intravascular clots stained in blue by PTAH. Images are representative of 3 (KO control) or 4 (the other groups) per group. PR8, influenza virus A/Puerto Rico/8/34; WT, wild type mice; KO, *Olr1* knockout mice; HE, hematoxylin and eosin; PTAH, phosphotungstic acid hematoxylin.

Pulmonary inflammation and thrombus formation in the lungs at the lethal phase of influenza were further investigated utilizing the severe influenza mouse model, and the results from WT mice were compared to those from KO mice (Fig. 6). No apparent difference was found in the microscopic observation of the lungs between WT and KO mice (Fig. 6a, b). The degree of lung inflammation caused by PR8 virus infection was examined in hematoxylin and eosin (HE)-stained sections from WT and KO mice sacrificed at 6 dpi when mice showed severe body weight losses. Lungs from both WT and KO mice demonstrated obvious peribronchial inflammation, inflammatory cells in alveoli, thickened alveolar walls, and alveolar hemorrhage after virus infection (Fig. 6c, d). Also, leukocytes in the vascular intima and perivascular spaces were observed in infected mice (Fig. 6c, d, inserts), suggesting activated leukocyte migration. No clear difference was observed between WT and KO mice. This result was compatible with serum cytokine data representing similar systemic cytokine secretion in KO mice to that in WT mice.

To confirm thrombus formation associated with severe influenza, phosphotungstic acid hematoxylin (PTAH) staining was performed, in which fibrin is stained in blue and thrombi can be visualized as well as fibrin deposition. As shown in Fig. 6e,f, increased intravascular thrombus formation in the lungs of WT mice infected with PR8 virus was indicated by multiple fibrin clots in blood vessels (Fig. 6e, arrows). In contrast to WT-infected mice, only sporadic thrombi were found in blood vessels of KO-infected mice similar to those in the uninfected groups, although HE staining indicated severe pulmonary inflammation in KO mice (Fig. 6f). Intravascular fibrin deposition observed in the lungs of infected KO mice seemed due to the clotting of residual blood in the blood vessels of the lungs after euthanasia, as they were found mainly in small veins, even in areas of less severe inflammation as in uninfected mice. The average numbers of clots [± standard error of the mean (SEM)] in the lung sections were 6.5 ± 2.7 in WT-control mice (n = 4), 53.8 ± 4.5 in WT-infected mice (n = 4), 7.7 ± 2.9 in KO-control mice (n = 3), and 9.8 ± 3.3 in KO-infected mice (n = 4). The number was significantly larger only in the WT infection group, compared to those in other groups (*p* < 0.0001, two-way ANOVA). These results demonstrated a critical role of OLR1 in severe influenza-induced intravascular thrombus formation in the lungs.

## Discussion

This study demonstrated prolonged PT and increased thrombin generation from prothrombin at the lethal phase of severe influenza in a mouse model. The results were consistent with previous findings in mouse and ferret influenza models^6,7^. Also, this study revealed a significant induction of aortic *Olr1* and its critical contribution to the thrombin generation and intravascular thrombosis in the lungs of mice with severe influenza. The important physiological roles of OLR1 have already been demonstrated in platelet activation, endothelial dysfunction, leukocyte migration, plaque formation, and atherosclerosis as a consequence of these pathological events^12,14,16,20^. In the context of acute coronary syndrome as a result of the progression of atherosclerosis, OLR1 has been thought to be involved in prothrombotic pathways induced by oxidized LDL^21^. Also, this study shows that OLR1 is critically involved in the first step of thrombus formation by promoting thrombin production in severe influenza. Although the detailed molecular mechanism needs to be elucidated, OLR1 induced in the vascular cells of mice infected with influenza virus may have promoted adhesion between platelets and the endothelial surface, as reported previously^16^, and increased thrombin generation by activated platelet. The contribution of OLR1 to PT prolongation in severe influenza was also demonstrated in this study. Suppose consumptive coagulopathy is the cause of PT prolongation as already suggested in a ferret influenza model^7^, the suppression of thrombin formation in KO mice would have reduced the consumption of coagulation factors, resulting in only mild PT prolongation.

Interestingly, increased intravascular fibrin clotting was not evident in other tissues of infected mice, e.g., the liver (data not shown), despite the elevation of circulating thrombin. Therefore, not only circulating thrombin but also factors associated with virus infection and/or a severe inflammatory response in the lung during influenza appear involved in a stable fibrin clot formation. For example, integrating previous reports on influenza and thrombus formation, increased expression of tissue factor, externalization of phosphatidylserine, and decreased blood flow velocity induced in the lungs by influenza virus infection are considered to promote thrombus formation preferably in the tissue^22–25^.

OLR1 expression is very low under normal conditions and upregulated in response not only to its ligand oxidized LDL^26^ but also various stimuli, such as lipopolysaccharide (LPS)^27–29^. Several transcription factors have been reported to regulate *Olr1* transcription. Given the genetic regulation of *Olr1* by inflammatory cascades through the activation of the transcription factor NF-κB^30,31^, influenza virus infection-induced systemic cytokine secretion could have induced *Olr1* in this study. A strong positive correlation between gene expression levels of *Olr1* and *Il6* suggested a link between OLR1 and inflammation. Because inflammation in the lungs and elevated blood cytokine levels in KO mice were similar to those in WT mice after virus infection, OLR1 does not regulate inflammatory responses but is a downstream factor induced by inflammation in the present experimental condition. The detailed mechanisms of *Olr1* induction by influenza virus infection remain elucidated. In addition to systemically secreted cytokines, of course, the possibility that the virus directly infects the cells of blood vessels^32^ and induces *Olr1* needs to be taken into account. However, given that *Olr1* is induced by various factors, its expression may have been induced by others aside from inflammatory molecules, such as oxidized LDL, angiotensin II, and metabolic abnormalities^26,33,34^, which have been previously reported to be induced during acute influenza^18,35,36^. Further studies on the mechanisms of *Olr1* induction by a viral infection will provide insights into biological responses to and pathogenesis of infectious diseases far beyond just cytokine induction.

Influenza virus infection-induced lung inflammation and systemic cytokine secretion were not affected by the absence of OLR1 in this study. However, OLR1 was involved in endotoxin-induced acute lung inflammation in a previous study in which an anti-OLR1 antibody pretreatment completely blocked immune cell activation and infiltration into the lungs after intraperitoneal injection with endotoxin^15,37^. This difference may reflect a pathophysiological difference between the host response to endotoxin and that to virus infection. In septic models, the administered LPS binds to Toll-like receptor (TLR) 2/4 on the cell membrane and causes an inflammatory response by activating NF-κB signaling in each cell in the first step. OLR1 has been reported to colocalize and cooperate with TLR2 to activate inflammatory responses by the outer membrane protein A of Gram-negative bacteria^38^. Furthermore, OLR1 functions as a bacterial receptor that enhances the adhesion of Gram-negative and Gram-positive bacteria to cells^39^. Therefore, because OLR1 is involved in the very early steps of TLR-mediated signaling on the cell membrane, the absence of OLR1 and its blockade may have strongly suppressed the inflammatory responses in the septic model. In contrast, during viral infection, virus entry into cells occurs first, and various viral molecules, such as viral membrane glycoproteins, viral constituent proteins, and nucleic acids, activate inflammation-related transducing cascades inside the infected cells, leading to the activation of NF-κB and other transcription factors to promote cytokine production^40^. Especially in the case of the influenza virus, at least two pathways thought to be independent of TLR2/4 have been reported to activate NF-κB: (1) endoplasmic reticulum stress induced by the overload of viral protein hemagglutinin^41^ and (2) double-stranded RNA-activated protein kinase^42^. Therefore, cytokine production in viral infection could be activated independently on OLR1. WT and KO mice showed a similar degree of weight loss after virus infection. This may be due to anorexia caused by increased circulating cytokines^43^. When focusing on biological responses after cytokine induction, weight loss does not seem to be a good indicator of the severity of the disease.

In summary, the findings indicated that influenza virus infection induces *Olr1* gene expression in the vascular system to promote thrombin generation and resultant intravascular clotting in the lungs. Thus, OLR1 is a promising novel therapeutic target to suppress the prothrombotic state during severe influenza. In addition, thrombosis has been observed to occur in many viral infections and is thought to be involved in the symptoms and severity of the diseases, including coronavirus disease 2019^44^. The importance of OLR1 in thrombosis should be considered in a wide range of infectious diseases.

## Materials and methods

### Virus

Influenza virus A/Puerto Rico/8/34 (H1N1; PR8) was kindly provided by the National Institute of Infectious Diseases (Tokyo, Japan). The virus was propagated in 10-day-old embryonated chicken eggs at 35 °C for 48 h, and aliquots of collected allantoic fluids were stored at − 80 °C until further analysis.

### Mice

The *Olr1* KO mice B6.129P2-*Olr1*^*tm1Saw*^ (KO mice in this manuscript) were generated as previously reported^19^. Male C57BL/6 mice purchased from Hokudo (Sapporo, Japan) and KO mice kindly given by Dr. Sawamura were kept in a BSL-2 laboratory and a clean room, respectively, at the International Institute for Zoonosis Control, Hokkaido University, under standard laboratory conditions (room temperature 22 °C ± 2 °C, relative humidity 50% ± 10%) and a 12/12 h light/dark cycle. For infectious experiments, KO mice were transferred to a BSL-2 laboratory and kept during experiments. Mice were administered a standard CE-2 chow diet purchased from CLEA Japan (Sapporo, Japan) with water *ad libtum*. Experiments were performed on 9- to 14-week-old male mice.

### Virus infection and sample collection

Virus infection and sample collection was carried out as previously reported^18^. PR8 virus particles at 500 PFUs in 50 µL phosphate-buffered saline (PBS) or PBS only (control) were intranasally inoculated into mice under inhalation anesthesia with isoflurane. Body weight was monitored daily. At 1, 3, or 6 dpi, mice were euthanized by overdose of isoflurane followed by cervical dislocation, and their blood, liver, and aorta, samples were collected. Blood samples were incubated at room temperature for 1 h to clot and then centrifuged at 1000*g* for 20 min at 4 °C. Supernatants were collected as serum and stored at − 20 °C until further analysis. Tissue samples were stored in TRIzol reagent (Thermo Fisher Scientific, Waltham, MA, USA) at − 80 °C until further analysis. This study was carried out in compliance with the ARRIVE (Animal Research: Reporting of In Vivo Experiments) guidelines except for blinding. Investigators could not be blinded because genetically modified animals because of the obligation to clearly indicate on the cage cards the treatment of animals, including viral infection, and the genetic modification information of the animals.

### Measurement of coagulation parameters

Whole-blood samples collected from mice were immediately used for PT-INR measurement with a CoaguChek Pro II (Roche Diagnosis, Mannheim, Germany) using a PT test strip. The concentrations of prothrombin fragment 1 + 2 in thawed serum samples were measured with a Mouse Prothrombin Fragment 1 + 2 ELISA kit (LS Bio, Seattle, WA, USA) according to the manufacturer’s instructions. Serum samples were diluted tenfold in a sample dilution reagent provided by the kit.

### Measurement of selected gene expression using real-time polymerase chain reaction (PCR)

Total RNA was extracted from tissue samples using TRIzol and was used for cDNA synthesis using High-Capacity cDNA Reverse Transcription Kits (Thermo Fisher Scientific) according to the manufacturer’s instructions. *Il6* (Mm00446190_m1), *Icam1* (Mm00516023_m1), and *Olr1* (Mm00454582_m1) gene expression was quantified using real-time PCR with a StepOne Real-Time PCR System (Applied Biosystems, Foster City, CA, USA) with indicated TaqMan probes (Applied Biosystems). The obtained gene expression was normalized to *18S* (Mm03928990_g1) from the same samples, and relative expression was calculated using the comparative Ct method (ΔΔCt).

### Measurement of the serum levels of cytokines and chemokines

Measurement of cytokines and chemokines in serum samples was carried out as previously reported^18^. The serum levels of IL-6, IP-10, MCP-1, and MIP-1β were determined using a MAGPIX Milliplex kit (Merck, Darmstadt, Germany) according to the manufacturer’s instructions. Briefly, 25 μL serum samples, standards, and controls were added to a 96-well plate comprising an equal amount of assay buffer for serum samples or serum matrix for standards and controls. Next, magnetic beads coated with antibodies against the target cytokines were added to each well, and the plates were incubated on a plate shaker overnight at 4 °C. After washing with washing buffer in the kit, the samples were reacted with biotinylated detection antibodies for 1 h and then with streptavidin–phycoerythrin for 30 min. After washing and the addition of loading buffer from the kit, the samples were analyzed by the MAGPIX system (Luminex, Austin, TX, USA).

### Measurement of lung viral titers

Mice were euthanized at 3 and 6 dpi, and their lung samples were collected and homogenized in 1 mL RPMI-anti medium [RPMI-1640 (Thermo Fisher Scientific) with 100 U/mL penicillin (Sigma-Aldrich), 100 µg/mL streptomycin (Sigma-Aldrich), and 20 µg/mL gentamicin (Thermo Fisher Scientific)]. After centrifugation at 3,000 rpm for 10 min, the supernatants were collected and stored at − 80 °C until further analysis. For plaque assays, monolayers of MDCK cells were prepared by seeding 1.2 × 10^6^ cells in 3 mL RP10 medium (RPMI-1640) supplemented with 10% inactivated fetal bovine serum (GE Healthcare UK Ltd., Little Chalfont, Buckinghamshire, UK), 1 mM sodium pyruvate (Thermo Fisher Scientific), 50 µM 2-mercaptoethanol (Merck), 100 U/mL penicillin, 100 µg/mL streptomycin, and 20 µg/mL gentamicin in each well of the tissue culture six-well plate and incubated overnight at 37 °C in 5% CO_2_. The monolayers were washed with RPMI-anti, and 125 µL tenfold serially diluted lung lysates were added to each well. The viruses were allowed to adsorb to the monolayers for 45 min, with shaking of the plates at 15 min intervals. Then, 3 mL prewarmed overlay medium consisting of Leibovitz L-15 with glutamine at pH 6.8 (Thermo Fisher Scientific) supplemented with 0.028% (w/v) NaHCO_3_ (Merck), 100 IU/mL penicillin, 100 mg/mL streptomycin, 0.1% (w/v) TPCK-treated trypsin (Merck), and 0.9% (w/v) agarose (BD Biosciences, Franklin Lakes, NJ, USA) were added to each well. The plates were then incubated at 37 °C in 5% CO_2_ for 3 days. Plaques on the monolayers were then counted without staining.

### Histopathological analyses

At 1, 3, or 6 dpi, mice were euthanized, and their lung samples were collected, immersion fixed in 4% paraformaldehyde, embedded in paraffin, and cut in 5 μm. Slides were stained with HE or PTAH after dewaxing in xylene and rehydration in decreasing ethanol concentrations. HE-stained lung sections were microscopically evaluated to assess the character and severity of pathologic lesions. Fibrin deposition and clotting were evaluated in PTAH-stained sections.

### Ethical statement

All mouse experiments were performed with approval (approval# 17-003) from the Animal Care and Use Committee of Hokkaido University following the Fundamental Guidelines for Proper Conduct of Animal Experiment and Related Activities in Academic Research Institutions under the jurisdiction of the Ministry of Education, Culture, Sports, Science and Technology in Japan. Body weight losses were monitored daily after infection, and mice were humanely euthanized when weight loss reached 25%.

### Statistical analysis

Statistical analyses were performed using Prism 7 (GraphPad Software, San Diego, CA, USA). Differences were identified using two-way ANOVA with a correction for multiple comparisons if necessary and considered significant when *p* < 0.05. Data are the mean ± SEM.

## Supplementary Information

Supplementary Information.
