# Supplementary material for: Critical role of oxidized LDL receptor-1 in intravascular thrombosis in a severe influenza mouse model
Source: Sci Rep. 2021 Aug 3;11:15675. doi: 10.1038/s41598-021-95046-y (PMC8333315; doi:10.1038/s41598-021-95046-y)
Supplement: Supplementary file 1 — Supplementary Information. [file 41598_2021_95046_MOESM1_ESM.pdf]

Title: Critical role of oxidized LDL receptor-1 in intravascular thrombosis in a severe influenza mouse model

Marumi Ohno, Akemi Kakino, Toshiki Sekiya, Naoki Nomura, Masashi Shingai, Tatsuya Sawamura, Hiroshi Kida

Supplemental Figure S1. Effect of OLR1 on *Il6* expression in the aorta and lungs

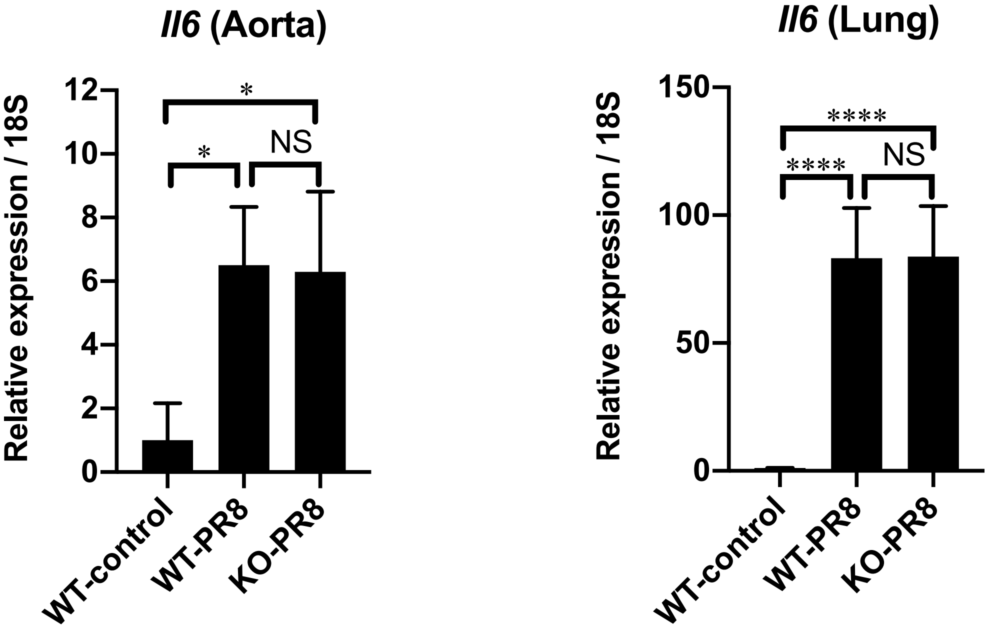

WT and KO mice were intranasally inoculated with PBS control or PBS comprising PR8 virus, and aortic and lung samples were collected at 6 dpi. *Il6* gene expression was normalized with that of 18S from real-time PCR analyses. Gene expression of PR8 virus-infected mice is presented as the fold changes relative to those of WT-control mice. Bars represent the mean ± SEM of four animals. White and black bars indicate data from control and PR8 virus-infected mice, respectively. * $p < 0.05$, ****$p < 0.0001$, two-way ANOVA using a multiple-comparison correction. PR8, influenza virus A/Puerto Rico/8/34; WT, wild type mice; KO, *Olr1* knockout mice; dpi, days post-infection; NS, not significant.
